# Intensive follow-up strategies after radical surgery for nonmetastatic colorectal cancer: A systematic review and meta-analysis of randomized controlled trials

**DOI:** 10.1371/journal.pone.0220533

**Published:** 2019-07-30

**Authors:** Yaqin Zhao, Cheng Yi, Yu Zhang, Fang Fang, Andrew Faramand

**Affiliations:** 1 West China Hospital, Sichuan University, Chengdu, Sichuan, China; 2 Affiliated Hospital of Chengdu University, Chengdu, Sichuan, China; 3 University of Pittsburgh Medical Center, Pittsburgh, Pennsylvania, United States of America; University of Mississippi Medical Center, UNITED STATES

## Abstract

**Background:**

Intensive follow-up after surgery for colorectal cancers is common in clinical practice, but evidence of a survival benefit is limited.

**Objective:**

To conduct a systematic review and meta-analysis on the effects of follow-up strategies for nonmetastatic colorectal cancer.

**Data sources:**

We searched Medline, Embase, and CENTRAL databases through May 30, 2018.

**Study selection:**

We included randomized clinical trials evaluating intensive follow-up versus less follow-up in patients with nonmetastatic colorectal cancer.

**Interventions:**

Intensive follow-up

**Main outcomes measures:**

Overall survival.

**Results:**

The analyses included 17 trials with a total of 8039 patients. Compared with less follow-up, intensive follow-up significantly improved overall survival in patients with nonmetastatic colorectal cancer after radical surgery (HR 0.85, 95% CI 0.74–0.97, P = 0.01; I2 = 30%; high quality). Subgroup analyses showed that differences between intensive-frequency and intensive-test follow-up (P = 0.04) and between short interval and long interval of follow-up (P = 0.02) in favor of the former one.

**Limitations:**

Clinical heterogeneity of interventions

**Conclusions:**

For patients with nonmetastatic colorectal cancer after curative resection, intensive follow-up strategy was associated with an improvement in overall survival compared with less follow-up strategy. Intensive-frequency follow-up strategy was associated with a greater reduction in mortality compared with intensive-test follow-up strategy.

## Introduction

Colorectal cancer is estimated to have affected 140 250 patient in 2018 in the United States. [1] It is the third most common cancer, and the second most common cause of cancer-related deaths. Total tumor resection remains the primary management option in patients with non-metastatic disease. However, the appropriate surveillance strategy after primary surgery is poorly defined. Some have argued for a more intensive follow up for early detection of tumor recurrence or metachronous disease.[2] However, others have argued against the need for intensive follow-up.[3] Additionally, the costs of the different follow-up strategies vary, range from hundreds to several thousands of dollars per patient, thus influencing the implementation of the different follow up strategies.[4]

Current follow up guidelines following complete resection vary. Most agree that these patients should at least undergo 5 year surveillance with computed tomography (CT scan, carcinoembryonic antigen testing (CEA), and lower GI endoscopy.[5–7] Individual trials[8, 9] have generally been underpowered; with the last two systematic reviews[10, 11] failing to show any survival benefit from intensive follow-up. Conclusions are though limited by the small sample sizes, relatively short follow-up, and modest trial quality. More recently, the results of a new randomized trial—the COLOFOL (A Pragmatic Study to Assess the Frequency of Surveillance Tests After Curative Resection in Patients With Stage II and III Colorectal Cancer) trial which was the largest trial on this topic—along with the results of the updated analysis of the FACS (Follow-up After Colorectal Surgery) trial and the GILDA (Gruppo Italiano Lavoro per la Diagnosi Anticipata) trial which were the second and the third largest trials, respectively, changed the landscape of evidence.[8, 12, 13] Thus, we performed an updated, systematic review of randomized clinical trials to determine the effects of intensive follow-up among patients with nonmetastatic colorectal cancer.

## Methods

### Protocol and guidance

This systematic review was conducted according to the protocol which was registered in PROSPERO database (CRD42018100574). We used established methods recommended by the Cochrane Handbook for Systematic Reviews of Interventions[14] to conduct the meta-analysis and reported the findings according to the Preferred Reporting Items for Systematic Reviews and Meta-Analyses(PRISMA) [15].

### Eligibility criteria

Eligible studies met the following PICOS criteria:

1. Population: nonmetastatic colorectal cancer patients of any age who have been treated with curative surgery.
2. Intervention: intensive follow-up strategies with control follow-up regimens, which were defined by the individual studies according to the diagnostic tests and frequency of monitoring.
3. Comparison intervention: less follow-up regimens, which was defined by the individual trials
4. Primary Outcome: overall survival (measured from the time of randomization in the study). Secondary outcomes were cancer-specific survival, relapse-free survival, salvage surgery, and interval recurrences.
5. Study design: randomized controlled trial.

Trials including patients with advanced cancer (e.g. Dukes’ stage D), when curative resection is generally not possible, where excluded.

### Information sources and search strategy

The search strategy was developed and executed with an experienced research librarian (PX) and was independently peer-reviewed by another investigator (YC). Medline, Embase, the Cochrane Central Register of Controlled Trials were searched electronically from inception until June 30, 2018, update on June 5, 2019. A second librarian independently peer-reviewed the search strategy. We consulted ClinicalTrials.gov and European Union Clinical Trials Register for ongoing studies and those completed with reported results. We also searched conference proceedings from American Society for Clinical Oncology, European Society for Therapeutic and Radiation Oncology, International Journal of Radiation Oncology Biology Physics: proceedings of the American Society for Radiation. Database searches were supplemented by screening the reference lists of relevant trials and reviews. No language restrictions were imposed. Details of the search strategy are presented in S1 File.

### Study selection

Two independent investigators (Yaqin Zhao and Yu Zhang) screened the titles and abstracts of reports. They screened the full text for potentially relevant studies when both agreed that a citation met the eligibility criteria. Disagreements between the investigators were resolved by consultation with a third investigator (FF). We contacted study authors to obtain missing information and unpublished data when needed to assess the inclusion criteria or when suitable data were not available.

### Data collection process

Two independent investigators (Yaqin Zhao and Yu Zhang) extracted data in duplicate using a pre-piloted standardized data-form and created tables for the evidence and outcomes. Articles reporting on the same trial at different follow-up timepoints were considered as a single trial for all analyses. Disagreements between the two investigators were resolved by consultation with a third investigator (FF).

### Assessment of risk of bias and quality of evidence

Two independent investigators (Yaqin Zhao and Yu Zhang) performed risk assessment following the approach in the Cochrane Handbook for Systematic Reviews of Interventions[16]. We assessed the following domains for each study: sequence generation, allocation concealment, blinding, incomplete outcome data, selective outcome reporting and other bias. We classified trials at high risk of bias if at least one domain was high risk. To evaluate the quality (certainty) of evidence for each outcome, we used the Grading of Recommendations, Assessment, Development and Evaluation (GRADE) guidance to assess the overall risk of bias, inconsistency, imprecision, indirectness and publication bias and summarized results in an evidence profile. [17]

### Data synthesis

We used RevMan 5.3.3 software (a freeware available from The Cochrane Collaboration) to conduct all analyses. All pooling analyses were done using random-effect model regardless of the level of heterogeneity because the included trials varied in clinical and methodological features. Time-to-event outcomes are most appropriately analyzed using hazard ratio (HR). For dichotomous outcomes, we calculated the odds ratio (OR) with 95% CI. We assessed heterogeneity using the Chi^2^ test (threshold p = 0.10), which was quantified using the I^2^ test (I^2^ > 50% being substantial). A P-value <0.05 was set for statistical significance. We planned to use a funnel plot to explore the possibility of publication bias when 10 or more trials were pooled.[18]

We conducted trial sequential analysis (TSA) for primary outcomes to explore whether cumulative data were adequately powered to evaluate outcomes.[19] An optimal information size set to a 2-sided 5% significance, 80% power, relative risk reduction of 20%, and the pooled control-group event rate across the included studies.

We prospectively identified 2 variables for subgroup analyses based on the main controversies in the debate: how frequently and what tests should patients be followed up with. We planned subgroup analyses for primary outcome including the following: 1. intensive follow-up strategy (intensive-frequency follow-up and intensive-test follow-up). Intensive-frequency follow-up was defined as more frequentl intensive follow-up with the same tests; intensive-test follow-up was defined as intensive follow-up with more types of tests at the same frequency. 2. intensive follow-up strategy on frequency of follow-up (short interval≤3 months and long interval >3 months) 3. intensive follow-up strategy on using CT (CT and no CT); 4. intensive follow-up strategy on using CEA (CEA and no CEA).

We perform post-hoc meta-regression based on frequency of follow-up, length of follow-up, mean age, and Dukes’ stage.

We planned sensitivity analyses by performing meta-analyses of results after removing 1 study at a time and removing earlier studies (before 2000).

## Results

### Study selection and study characteristics

**Fig 1:**
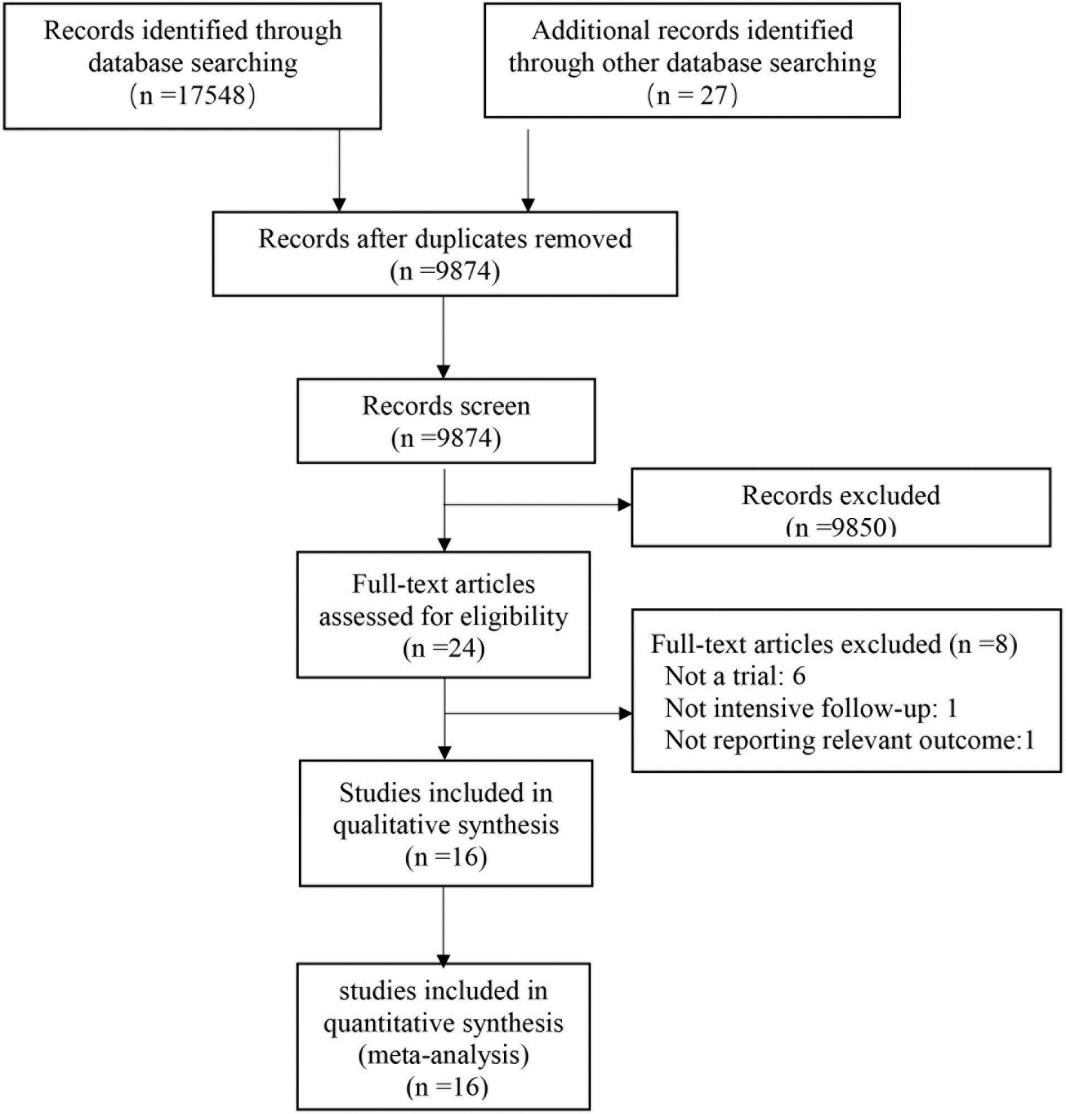
Flow diagram of the literature search.

The results of the literature search are shown in Fig 1. Of the 17575 results, 24 pertinent studies were identified and included in full-text review. After review, 8 studies were excluded because they did not meet the inclusion criteria (S1 File).[20–27] A total of 16 trials were selected for the present analysis, including a total of 7908 patients.[8, 12, 13, 28–40] The kappa for systematic searches, selection of studies and data extraction were 1.00, 0.89 and 0.97, respectively.

Table 1 shows the main characteristics of selected trials. The trials were published between 1995 and 2018. Population sizes ranged from 106 to 2555. Seven trials[12, 30, 31, 33, 34, 38, 39] compared intensive follow-up strategy with less follow-up strategy, whereas 4 trials[13, 32, 35, 36] compared intensive follow-up group with no/minimal any follow-up. The frequency of the follow-up strategy in the included trials varied. Most of the trials carried out follow-up every 3 months for 2 years, then every 6 months for 3 years.

**Table 1:** The characters of included trials.

Author	Year	No patients	Country	Dukes’ stage	Intervention group	Control group	Follow-up (months)
Augestad	2013	110	Norway	Dukes' A: 24; Dukes' B: 55; Dukes' C: 32	Surgeon follow-up	GP follow-up	24
COLOFOL	2018	2555	Sweden	Unclear	CT (thorax and abdomen) and CEA at 6, 12, 18, 24, and 36 months	CT (thorax and abdomen) and CEA at 12 and 36 months	60
GILDA	2016	1228	Italy, Spain, US	Dukes' B: 617; Dukes' C: 611	4, 8, 12, 16, 20, 24, 30, 36, 42, 48, and 60 monthly office visits and history and clinical examination, FBC, CEA, and CA 19–9; Colonoscopy and CXR at 12, 24, 36, 48, and 60 months; Liver ultrasound at 4, 8, 12, 16, 24, 36, 48, and 60 months; For rectal participants, pelvic CT at 4, 12, 24, and 48 months	4, 8, 12, 16, 20, 24, 30, 42, 48, and 60 monthly office visits, including history, examination, and CEA; Colonoscopy at 12 and 48 months; Liver ultrasound at 4 and 16 months; Rectal cancer participants in addition had rectoscopy at 4 months, CXR at 12 months, and liver US at 8 and 16 months. A single pelvic CT was allowed if a radiation oncologist required it as baseline following adjuvant treatment	96
Kjeldsen	1997	597	Denmark	Dukes' A 138; Dukes' B: 293; Dukes' C: 166	At 6, 12, 18, 30, 36, 48, 60, 120, 150 and 180 months, digital rectal examination, colonoscopy, CXR: the same in both groups.	At 60, 120, 180 months, digital rectal examination, colonoscopy, CXR: the same in both groups.	132
Mäkelä	1995	106	Finland	Dukes' A: 28; Dukes' B: 48; Dukes' C: 30	participants who had rectal or sigmoid cancers had flexible sigmoidoscopy with video imaging every 3 months, colonoscopy at 3 months (if it had not been done pre-operation), then annually. They also had ultrasound of the liver and primary site at 6 months, then annually.	participants who had rectal and sigmoid cancers had rigid sigmoidoscopy and barium enema annually	60
Ohlsson	1995	107	Finland	Dukes' A: 19; Dukes' B: 47; Dukes' C: 41	at 3-, 6-, 9-, 12-, 15-, 18-, 21-, 24-, 30-, 36-, 42-, 48-, and 60-month intervals. Performed at each visit were clinical exam, rigid proctosigmoidoscopy, CEA, alkaline phosphatase, gamma-glutaryl transferase, faecal haemoglobin, and CXR. Examination of anastomosis (flexible sigmoidoscopy or colonoscopy, as dictated by the lesion) was performed at 9, 21, and 42 months. Colonoscopy was performed at 3, 15, 30, and 60 months. CT of the pelvis was performed at 3, 6, 12, 18, and 24 months.	no follow-up visits planned. They received written instructions recommending that they leave faecal samples with the district nurse for examination every third month during the first 2 years after surgery then once a year. They were instructed to contact the surgical department if they had any symptoms.	66–105.6
Pietra	1998	207	Italy	Dukes' A: 0; Dukes' B: 122; Dukes' C: 85	At 3, 6, 9, 12, 15, 18, 21, 24, 30, 36, 42, 48, 54, and 60 months, then annually thereafter. At each visit, clinical examination, ultrasound, CEA, and CXR were performed. Annual CT and colonoscopy were performed.	At 6 and 12 months, then annually. At each visit, clinical examination, CEA, and ultrasound were performed. Annual CXR, colonoscopy, and CT were performed.	60
FACS	2017	1202	UK	Dukes' A: 254; Dukes' B: 553; Dukes' C: 354	(1) CEA follow-up: measurement of blood CEA every 3 months for 2 years, then every 6 months for 3 years, with a single chest, abdomen, and pelvis CT scan at 12–18 months if requested at study entry by hospital clinician (n = 300). (2) CT follow-up: CT of the chest, abdomen, and pelvis every 6 months for 2 years, then annually for 3 years (n = 299). (3) CEA and CT follow-up: both blood CEA measurement and CT imaging as above (n = 302).	no scheduled follow-up except a single CT scan of the chest/abdomen/pelvis if requested at study entry by a clinician	106
Rodriguez-Moranta	2006	259	Spain	Unclear	Seen with history, examination, and bloods (including CEA) at 3, 6, 9, 12, 15, 18, 21, 24, 27, 30, 33, 36, 39, 42, 45, 48, 51, 54, 57, and 60 months. US/CT at 6, 12, 18, 24, 30, 36, 42, 48, and 56 months. CXR and colonoscopy at 12, 24, 36, 48, and 56 months	Seen with history, examination, and bloods (including CEA) at 3, 6, 9, 12, 15, 18, 21, 24, 27, 30, 33, 36, 39, 42, 45, 48, 51, 54, 57, and 60 months	48
Schoemaker	1998	325	Australia	Dukes' A: 71; Dukes' B: 153; Dukes' C: 101	Participants in the experimental arm underwent yearly CXR, CT of the liver, and colonoscopy.	These investigations were only performed in the control group if indicated on clinical grounds or after screening test abnormality, and at 5 years of follow-up, to exclude a reservoir of undetected recurrences.	60
Secco	2002	227	Italy	Unclear	They had clinic visits and serum CEA, abdomen/pelvic US scans, and CXR. Participants with rectal carcinoma had rigid sigmoidoscopy and CXR.	Minimal follow-up programme performed by physicians	61.5 48
Sobhani	2008	130	French	Unclear	PET performed at 9 and 15 months and conventional follow-up	conventional follow-up	24
Strand	2011	110	Sweden	Unclear	surgeon-led follow-up	nurse-led follow-up	60
Treasure	2014	216	UK	Dukes' A: 10; Dukes' B: 95; Dukes' C: 74	Second-look laparotomy	No further action was taken	300
Wang	2009	326	China	Dukes' A: 53; Dukes' B: 186; Dukes' C: 93	Colonoscopy at 3-month intervals for 1 year, at 6-month intervals for the next 2 years, and once a year thereafter	Colonoscopy at six months, 30 months, and 60 months postoperatively	64–79
Wattchow	2006	203	Australia	Dukes' A: 47; Dukes' B: 96; Dukes' C: 60	Follow by surgeons: more ultrasound, colonoscopy and sigmoidoscopy. CEA, CT, Rx, endoscopy: the same in both groups.	Follow-up by general practitioners: more fecal occult blood. CEA, CT, Rx, endoscopy: the same in both groups.	24

### Risk of bias and quality of evidence

Risk of bias assessments are reported in S1 File. None trial was judged as low risk of bias, 11 were unclear risk, and 5 were high risk. Table 2 shows GRADE summary of all outcomes.

**Table 2:** Summary of findings and strength of evidence in studies of the effects of intensive follow-up among patients with nonmetastatic colorectal cancer.

Outcome	No. of patients (Studies)	Relative effect (95% CI)	I^2^	Absolute effect estimates (per 1000)			Strength of Evidence (GRADE)
				Less Follow-up	Intensive Follow-up	Difference	
Overall survival	7170 (15)	HR 0.85 (0.74 to 0.97)	36%	240	206	-34 (-7 to -59)	High
Colorectal survival	4003 (9)	HR 0.90 (0.77 to 1.04)	0%	112	105	-7 (-9 to 53)	High
Relapse-free survival	5359 (13)	HR 1.04 (0.94 to 1.16)	0%	113	120	7 (-11 to 27)	High
Salvage surgery	4558 (13)	OR 2.23 (1.59 to 3.12)	62%	62	128	66 (33 to 109)	Moderate^1^
Interval recurrences	5832 (8)	OR 0.72 (0.44 to 1.19)	82%	147	110	-37 (-76 to 23)	Low^1^^,^^2^

**CI:** Confidence interval; **HR:** Hazard ratio; OR: odds ratio

^1^ inconsistency

^2^ imprecisions

### Primary outcome: Overall survival

**Fig 2:**
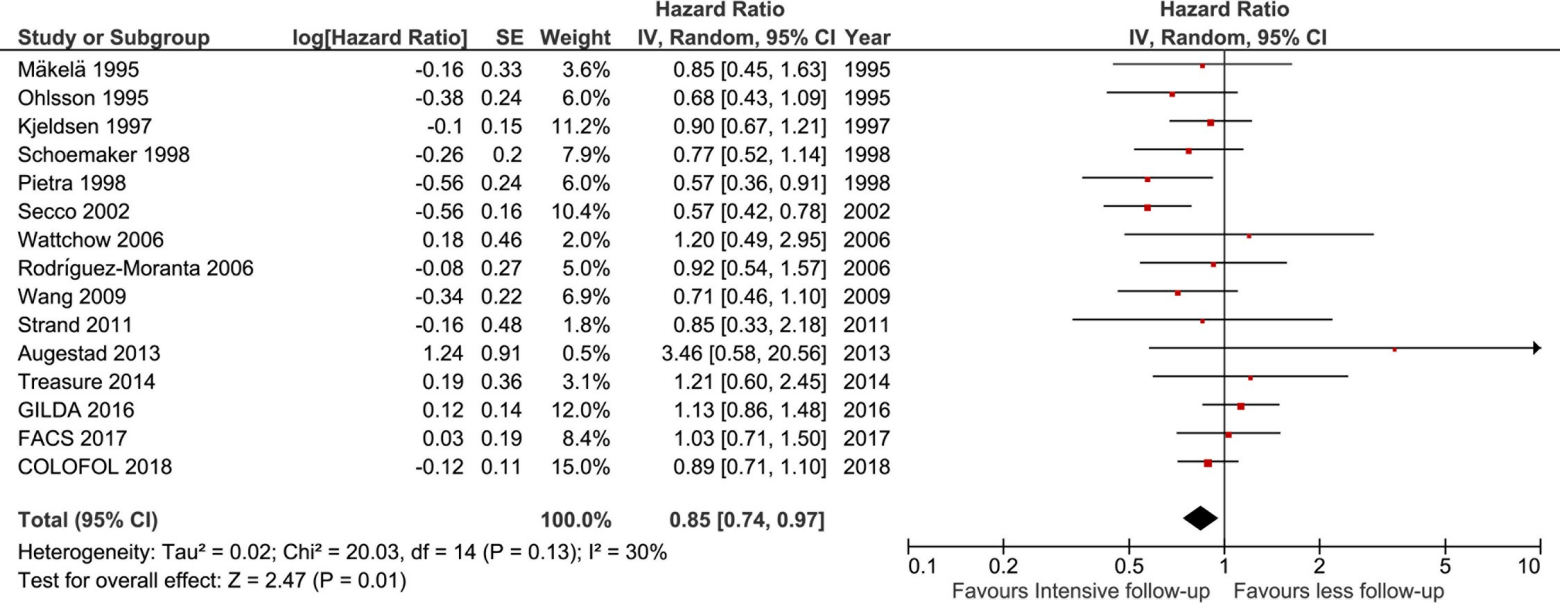
Forest plot of overall survival of all trials. df = degrees of freedom, M-H = Mantel-Haenszel.

**Fig 3:**
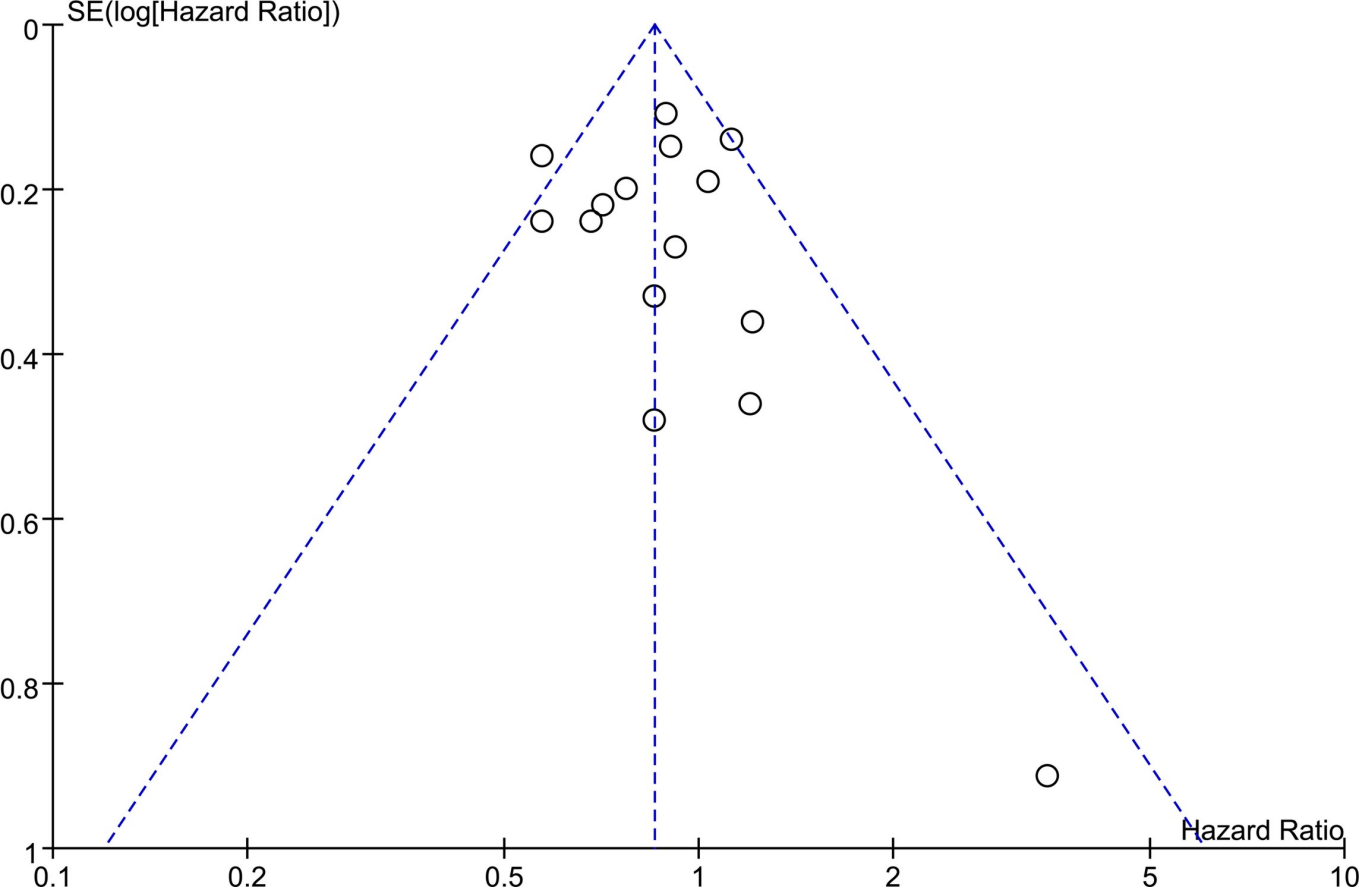
Funnel plot analysis of overall survival.

The associations between intensive versus less follow-up and overall survival are shown in Fig 2. Fifteen trials reported outcomes of all-cause mortality. Overall, all-cause mortality was 21.7% (784/3604) in the intensive follow-up group and 24.0% (857/3566) in the less follow-up group (OR 0.82, 95% CI 0.70–0.96; I^2^ = 29%). Forteen trials reported the data of time-to-event. Pooled HR showed a protective effect of an intensive follow-up strategy on overall survival (HR 0.84, 95% CI 0.74–0.96; I^2^ = 33%; high quality). Funnel plot analysis did not suggest any asymmetry (Fig 3), and the Egger test did not detect significant publication bias (P = .11). Moreover, TSA confirmed that the required information size was met (S1 File)

### Cancer-specific survival and relapse-free survival

Nine trials presented outcomes of cancer-specific survival. No significant differences were found between intensive and less follow-up in cancer-related survival (HR 0.90, 95% CI 0.77–1.04, I^2^ = 0%, S1 File). Fourteen trials provided data on relapse-free survival. Also, a pooled HR failed to show a significantly protective effect of intensive follow-up (HR 1.04, 95% CI 0.94–1.16, I^2^ = 0%).

### Salvage surgery and interval recurrences

Among 13 trials, salvage surgery occurred in 11.9% patients in intensive follow-up group and in 6.2% patients in less follow-up group. The odds of salvage surgery in patients receiving intensive follow-up was 2 times as high as that in patients receiving less follow-up (OR 2.23, 95% CI: 1.59–3.12, I^2^ = 45%, S1 File). Eight trials presented outcomes of interval recurrences. The meta-analysis showed no significant difference in interval recurrences according to the surveillance strategy (OR 0.72, 95% CI 0.44 to 1.19, I^2^ = 83%, S1 File)

### Subgroup analyses, meta-regression, and sensitivity analyses

Overall survival was significantly higher among trials with intensive-frequency follow-up strategy (HR 0.82, 95% CI 0.69–0.97; P for interaction = 0.04; Table 3 and S1 File) and among trials with short interval of follow-up (HR 0.75, 95% CI 0.64–0.87; P for interaction = 0.02; S1 File). Subgroup analyses based on type of tests (CT or CEA) revealed no significant interactions with study settings). Sensitivity analyses (removing 1 study at a time and removing early studies) showed similar results of overall survival (S1 File). Meta-regression showed no interaction based on frequency of follow-up, length of follow-up, mean age, and Dukes’ stage (S1 File).

**Table 3:** Subgroup analysis of the effect of intensive follow-up on overall survival.

Subgroup title	Trial	I^2^	HR (95% CI)	P
Intensive follow-up strategy				
Intensive frequency	4	17%	0.82 [0.69, 0.97]	0.04
Intensive test	4	0%	1.07 [0.88, 1.31]	
Frequency of follow-up				
Short interval(≤3 months)	9	11%	0.75 [0.64, 0.87]	0.02
Long interval (>3 months)	4	0%	0.96 [0.84, 1.11]	
Using CEA				
CEA	5	0%	0.97 [0.84, 1.13]	0.66
No CEA	1	-	0.90 [0.67, 1.21]	
Using CT				
CT	7	0	0.93 [0.82, 1.06]	0.57
No CT	2	53%	1.31 [0.40, 4.23]	

## Discussion

This meta-analysis demonstrates that, intensive follow-up significantly improved overall survival in patients with nonmetastatic colorectal cancer after radical surgery. Moreover, this meta-analysis suggested that intensive follow-up resulted in a significant increase in the odds of salvage surgery. Finally, this meta-analysis showed no evidence of effects of intensive follow-up on benefits of relapse-free survival or cancer-specific survival.

### Compared with other studies

Earlier reviews[41–43] on this topic suggested that intensive follow-up was associated with improved survival. Yet, contrary to our study, the last two reviews[10, 11] which were carried out in 2016 failed to replicate the benefits on survival. A Cochrane review, including a total of 15 trials randomizing 5403 patients after surgery for colorectal cancer, failed to identify credible effect on survival from intensive follow-up (HR 0.90; 95% CI 0.78–1.02). In parallel, an additional systematic review by Mokhles et al., which included 16 trials and 7081 patients, also failed to find a favorable effect on survival (HR 0.98; 95% CI 0.87–1.11).

Differences between our study and the last studies[10, 11] might be explained by our study including a recently published trail and two updated trial, which were the top 3 trials on this topic and accounted for 63.0% (4985/7908) of the total number of patients. Our meta-analysis has made it possible to provide improved precision concerning the effects of intensive follow-up and met minimum information size in TSA. Further, we quantified two new findings of subgroup analyses that frequently intensive follow-up has a reasonable survival profile in nonmetastatic colorectal cancer.

### Strengths and limitations

Strengths of this meta-analysis include a utilizing a published protocol in PROSPERO, a comprehensive search, duplicable assessment of eligibility, risk of bias, and data abstraction, the use of a more conservative random effects model, no restriction on language or time of publication for included trials, and the use of an outcome measure which incorporates the time-to-event nature of the data. Strengths of this study also include rigorous assessment of the quality of evidence (and found the quality for primary outcome high), of the TSA (and met the minimum information size), of the credibility of subgroup analyses (and identified crucial differences in frequency of intensive follow-up).

This study has limitations. First, the results of this meta-analysis were weakened by significant heterogeneity of definition of intervention across included trials, with a moderate degree of detected heterogeneity for the primary outcome (I^2^ = 30%), justifying the use of random-effects models. To explore the source of heterogeneity, we conducted several subgroup analyses. In subgroup analyses based on surveillance strategy (intensive-frequency follow-up and intensive-test follow-up), heterogeneity could be resolved, and significant subgroup difference was found.

Second, the protocol of including trials was developed several decades ago; since then, treatments and diagnostic techniques for colorectal cancer have evolved. For example, some trials were initiated in an era before the widespread use of the CEA blood test as a monitoring test for recurrence of colorectal cancer. However, specificity and sensitivity in detecting disease recurrence depends largely on the definition of abnormal CEA levels. However, the cutoff CEA varies in era and location.

Third, this review did not report adverse effects. Those were rare events, and thus this review was underpowered to evaluate the safety. Observational studies may be more appropriate than trials to evaluate the safety, as these often include more individuals and follow-up may be longer.

### Applicability

In practice, there was considerable variance in strategy for surveillance after surgery for colorectal cancer. [44, 45] The follow-up strategies vary in major societies. [5–7] There are also considerable differences in the costs of in the different health systems and reimbursed ways of the services, ranging from hundreds to several thousands of dollars per patient.[4, 46] The results of this study suggest that intensive follow-up for patients with colorectal cancer after curative surgery improves overall survival. Meta-analysis of subgroups investigating frequently intensive follow-up suggests a favorable effect on all-cause mortality. According to these findings, follow-up should be considered at every 3 months. Moreover, intensive follow-up strategies have potentially important resource and financial implications for health services. Application of economic analysis is beyond the scope of this study; however, the present study should serve as a basis for health economic modeling in future studies.

## Conclusion

Among patients with colorectal cancer after curative surgery, intensive follow-up strategy was associated with an improvement in overall survival. This benefit was observed in intensive-frequency follow-up strategy but not intensive-test follow-up strategy.

## Supporting information

S1 File Supplementary e-material. (DOCX)

S2 File PRISMA checklist. (DOC)
