# Supplementary material for: Intensive follow-up strategies after radical surgery for nonmetastatic colorectal cancer: A systematic review and meta-analysis of randomized controlled trials
Source: PLoS One. 2019 Jul 30;14(7):e0220533. doi: 10.1371/journal.pone.0220533 (PMC6667274; doi:10.1371/journal.pone.0220533)

**S1 File. Supplementary e-material**

|  |
| --- |
| eTable 1: Search strategy |
| eTable 2. List of excluded trials and reasons for exclusion |
| eTable 3: Sensitivity analyses for the primary outcome |
| eTable 4: Meta-regression for the primary outcome |
| eFigure 1: Risk of bias summary |
| eFigure 2: Trial sequential analysis of overall survival |
| eFigure 3: Forest plot of cancer-specific survival |
| eFigure 4: Forest plot of salvage surgery of all trials |
| eFigure 5: Forest plot of interval recurrences |
| eFigure 6: Subgroup analysis for overall survival –based on intensive follow-up strategy |
| eFigure 7: Subgroup analysis for overall survival –based on frequency of follow-up |
|  |
|  |
|  |

### Supplement eTable 1 Literature search strategy:

| **MEDLINE(R)** | |
| --- | --- |
| 1 | exp colorectal neoplasms/ |
| 2 | (colon tumor or colon cancer or colon carcinoma or colon adenocarcinoma or colorectal tumor or sigmoid carcinoma or rectum carcinoma or rectum cancer or rectum tumor or rectum adenoma or colorectal carcinoma or colorectal cancer).mp. |
| 3 | 1 or 2 |
| 4 | exp longitudinal studies or exp survival analysis or exp mortality or exp prognosis or exp population surveillance or exp treatment outcome |
| 5 | (recurrence or neoplasm recurrence or neoplasm metastasis or recurr$ or cancer survival or follow up or follow-up).mp. |
| 6 | 4 or 5 |
| 7 | randomized controlled trial.pt. or controlled clinical trial.pt. or randomized.ab. or placebo.ab or drug therapy.fs. or randomly.ab or trial.ab. or groups.ab. |
| 8 | exp animals/ not humans.sh |
| 9 | 7 not 8 |
| 10 | 3 and 6 and 9 |
| **Embase** | |
| 1 | exp colorectal neoplasms/ |
| 2 | (colon tumor or colon cancer or colon carcinoma or colon adenocarcinoma or colorectal tumor or sigmoid carcinoma or rectum carcinoma or rectum cancer or rectum tumor or rectum adenoma or colorectal carcinoma or colorectal cancer).af. |
| 3 | 1 or 2 |
| 4 | exp longitudinal studies/ or exp survival analysis/ or exp mortality/ or exp prognosis/ or exp population surveillance/ or exp treatment outcome/ |
| 5 | (recurrence or neoplasm recurrence or neoplasm metastasis or recurr$ or cancer survival or follow up or follow-up).af. |
| 6 | 4 or 5 |
| 7 | (randomized controlled trial or controlled clinical trial).pt. or randomized.ab. or placebo.ab. or drug therapy.fs. or randomly.ab. or trial.ab. or groups.ab. |
| 8 | exp animal/ |
| 9 | human/ |
| 10 | 8 not 9 |
| 11 | 7 not 10 |
| 12 | 3 and 6 and 11 |
| **Cochrane CENTRAL** | |
| 1 | exp colorectal neoplasms/ |
| 2 | (colon tumor or colon cancer or colon carcinoma or colon adenocarcinoma or colorectal tumor or sigmoid carcinoma or rectum carcinoma or rectum cancer or rectum tumor or rectum adenoma or colorectal carcinoma or colorectal cancer).af. |
| 3 | 1 or 2 |
| 4 | exp longitudinal studies/ or exp survival analysis/ or exp mortality/ or exp prognosis/ or exp population surveillance/ or exp treatment outcome/ |
| 5 | (recurrence or neoplasm recurrence or neoplasm metastasis or recurr$ or cancer survival or follow up or follow-up).af. |
| 6 | 4 or 5 |
| 7 | 3 and 6 |

### Supplement eTable 2. List of excluded trials and reasons for exclusion

| Trial | Year | Reason for exclusion |
| --- | --- | --- |
| Detry | 2001 | Not a trial |
| Meyerhardt | 2003 | Not a trial |
| Papagrigoriadis | 2007 | Not a trial |
| Renehan, | 2004 | Not a trial |
| Snyder | 2018 | Not a trial |
| Verberne | 2015 | Not reporting relevant outcome |
| Wichmann | 2002 | Not a trial |
| Zhang | 2014 | Not intensive follow-up |

Supplement eTable 3 sensitivity analyses for the primary outcomes (results after removing 1 study at a time and removing early studies).

| Removing | Year | HR (95%CI) |
| --- | --- | --- |
| [Augestad](http://cochranelibrary-wiley.com/doi/10.1002/14651858.CD002200.pub3/full#CD002200-bbs2-0001) | 2013 | 0.84 [0.74, 0.95] |
| GILDA | 2016 | 0.81 [0.71, 0.92] |
| [Kjeldsen](http://cochranelibrary-wiley.com/doi/10.1002/14651858.CD002200.pub3/full#CD002200-bbs2-0004) | 1997 | 0.83 [0.72, 0.97] |
| [Mäkelä](http://cochranelibrary-wiley.com/doi/10.1002/14651858.CD002200.pub3/full#CD002200-bbs2-0005) | 1995 | 0.84 [0.73, 0.97] |
| [Ohlsson](http://cochranelibrary-wiley.com/doi/10.1002/14651858.CD002200.pub3/full#CD002200-bbs2-0006) | 1995 | 0.85 [0.74, 0.98] |
| Pietra | 1998 | 0.86 [0.76, 0.98] |
| FACS | 2017 | 0.82 [0.71, 0.95] |
| Rodrig[uez-Moranta](http://cochranelibrary-wiley.com/doi/10.1002/14651858.CD002200.pub3/full#CD002200-bbs2-0008) | 2006 | 0.84 [0.73, 0.97] |
| Schoemaker | 1998 | 0.85 [0.73, 0.98] |
| Strand | 2011 | 0.84 [0.74, 0.96] |
| Secco | 2002 | 0.89 [0.80, 1.00] |
| Treasure | 2014 | 0.83 [0.73, 0.95] |
| Wang | 2009 | 0.85 [0.73, 0.98] |
| COLOFOL | 2018 | 0.83 [0.71, 0.97] |
| Early studies | Before 2000 | 0.84 [0.71, 1.00] |

Supplement eTable 4 Meta-regression analysis for the primary outcome

| Factors | P for interaction |
| --- | --- |
| frequency of follow-up | 0.07 |
| length of follow-up | 0.89 |
| mean age | 0.76 |
| Dukes’ stage | 0.54 |

### Supplement eFigure 1. Risk of bias summary: review authors' judgements about each risk of bias item for each included study.


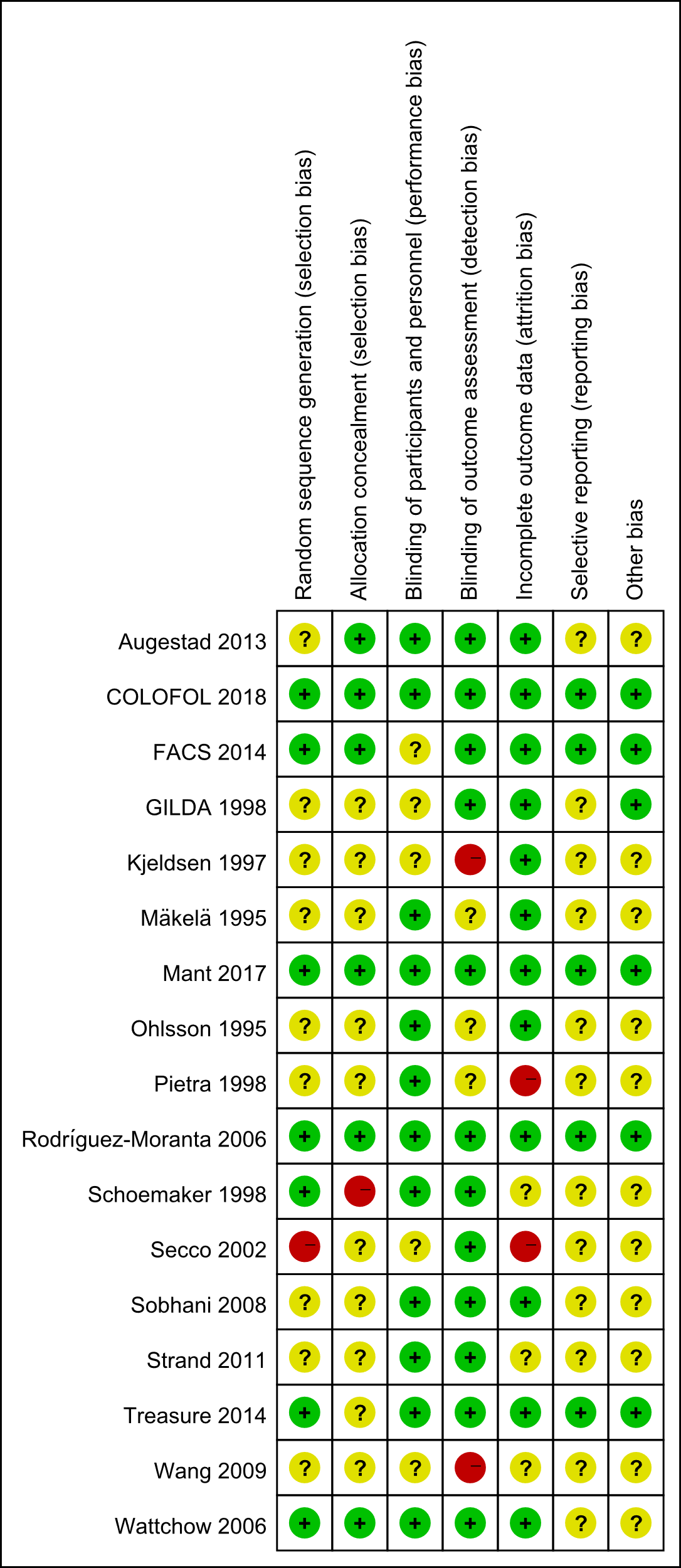


##### Supplement eFigure 2. Trial sequential analysis of overall survival


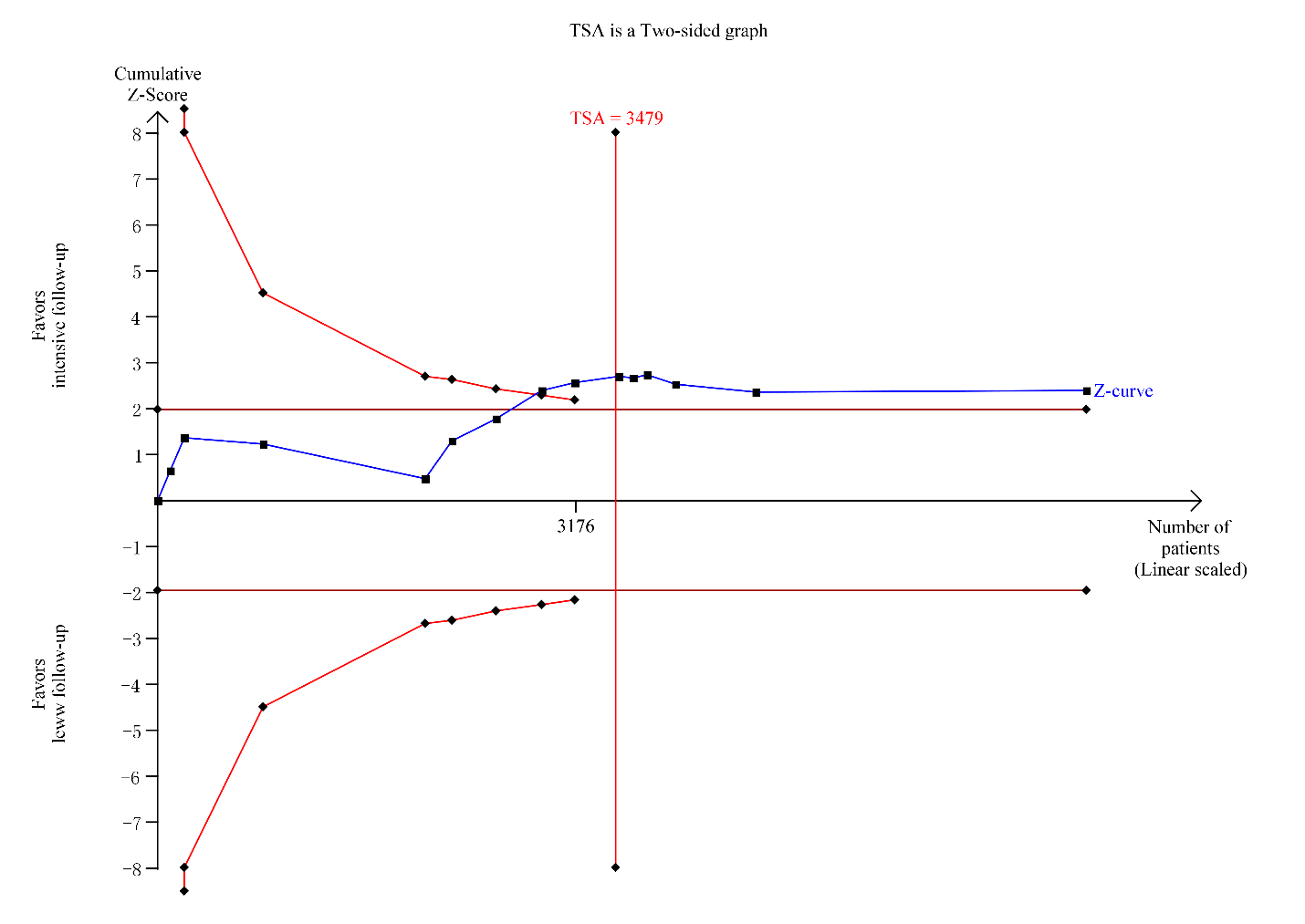


### Supplement eFigure 3. Forest plot of cancer-specific survival. df = degrees of freedom, M-H = Mantel-Haenszel.


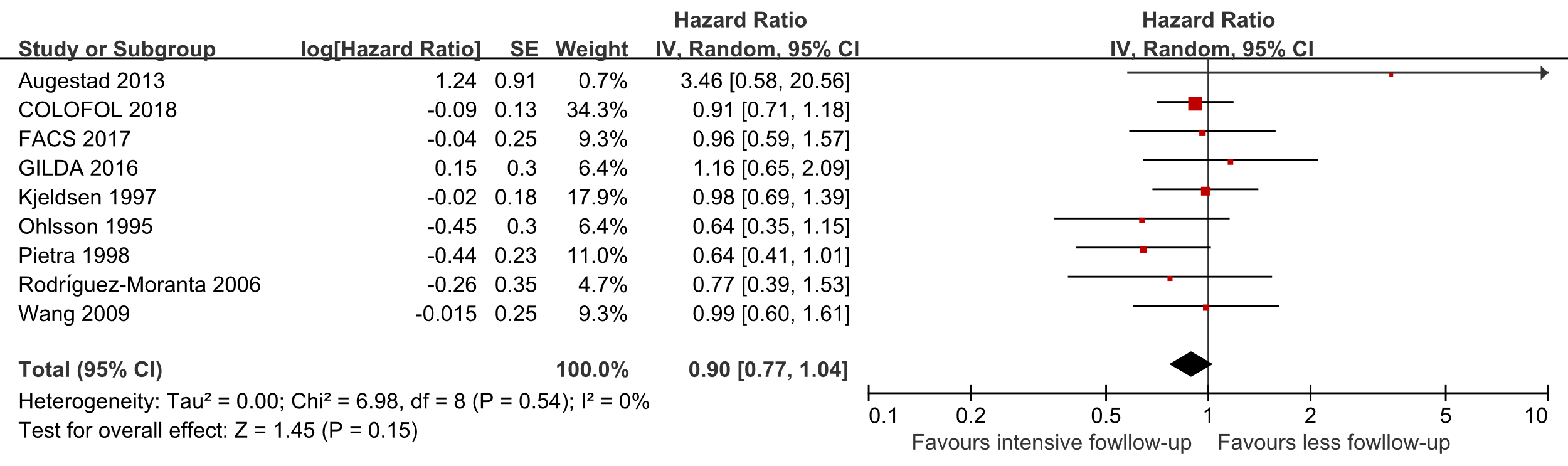


### Supplement eFigure 4: Forest plot of salvage surgery. df = degrees of freedom, M-H = Mantel-Haenszel.

###
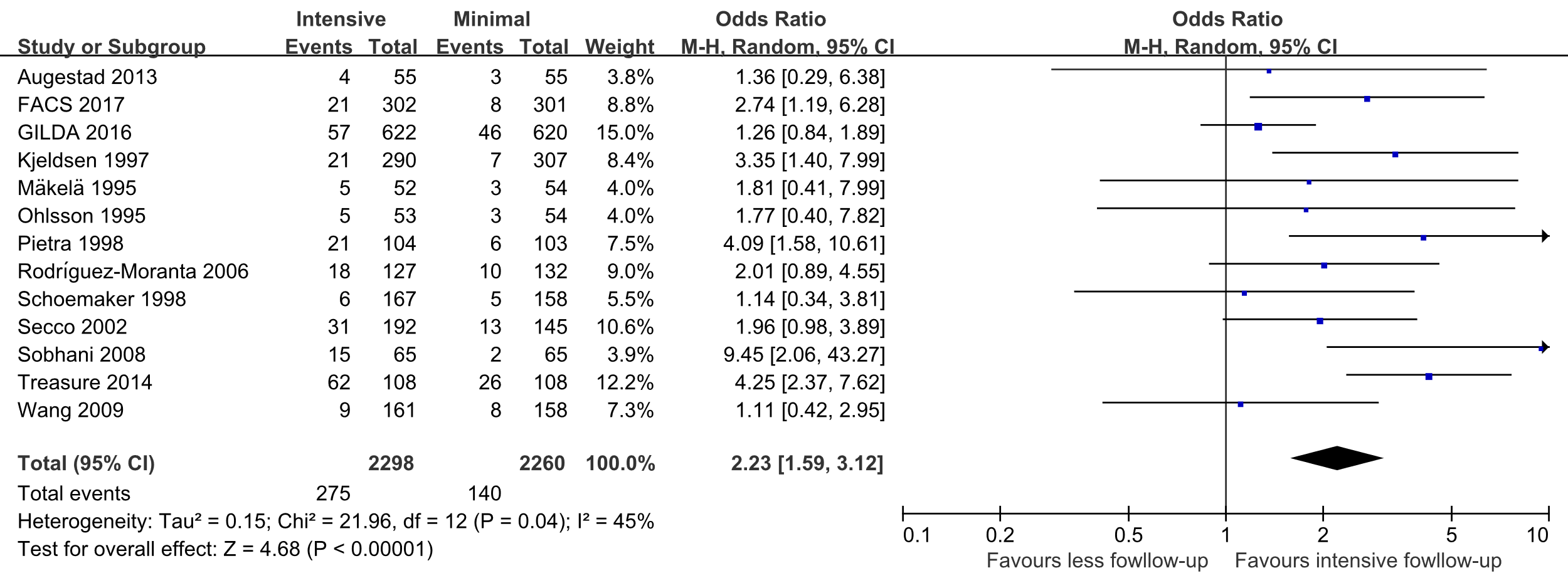


### Supplement eFigure 5: Forest plot of interval recurrences. df = degrees of freedom, M-H = Mantel-Haenszel.

###
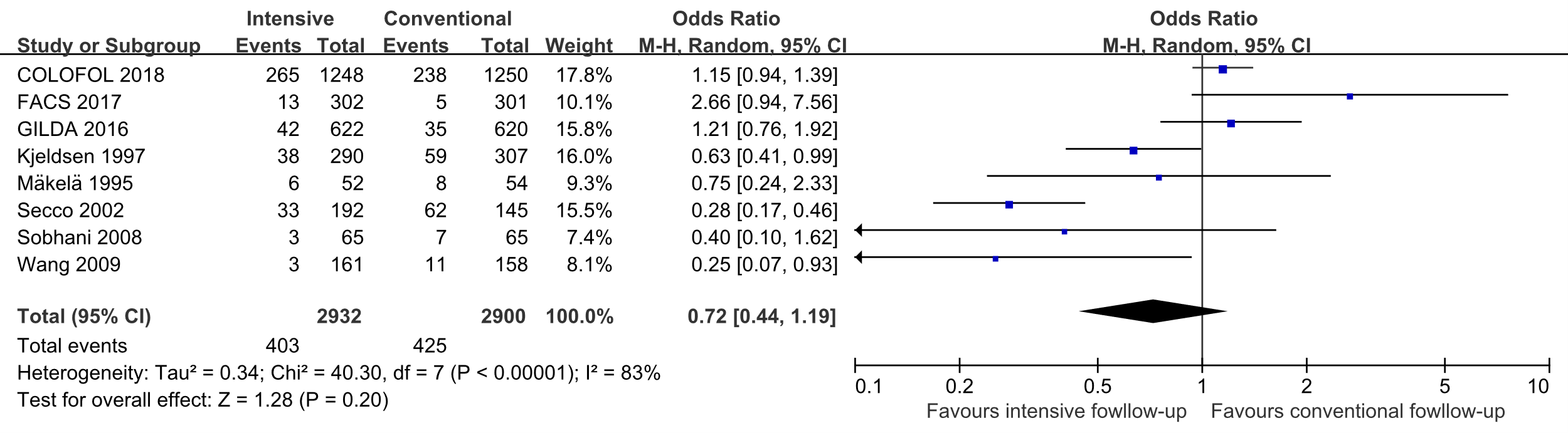


### Supplement eFigure 6: Subgroup analysis for overall survival –based on intensive follow-up strategy. df = degrees of freedom, M-H = Mantel-Haenszel.

###
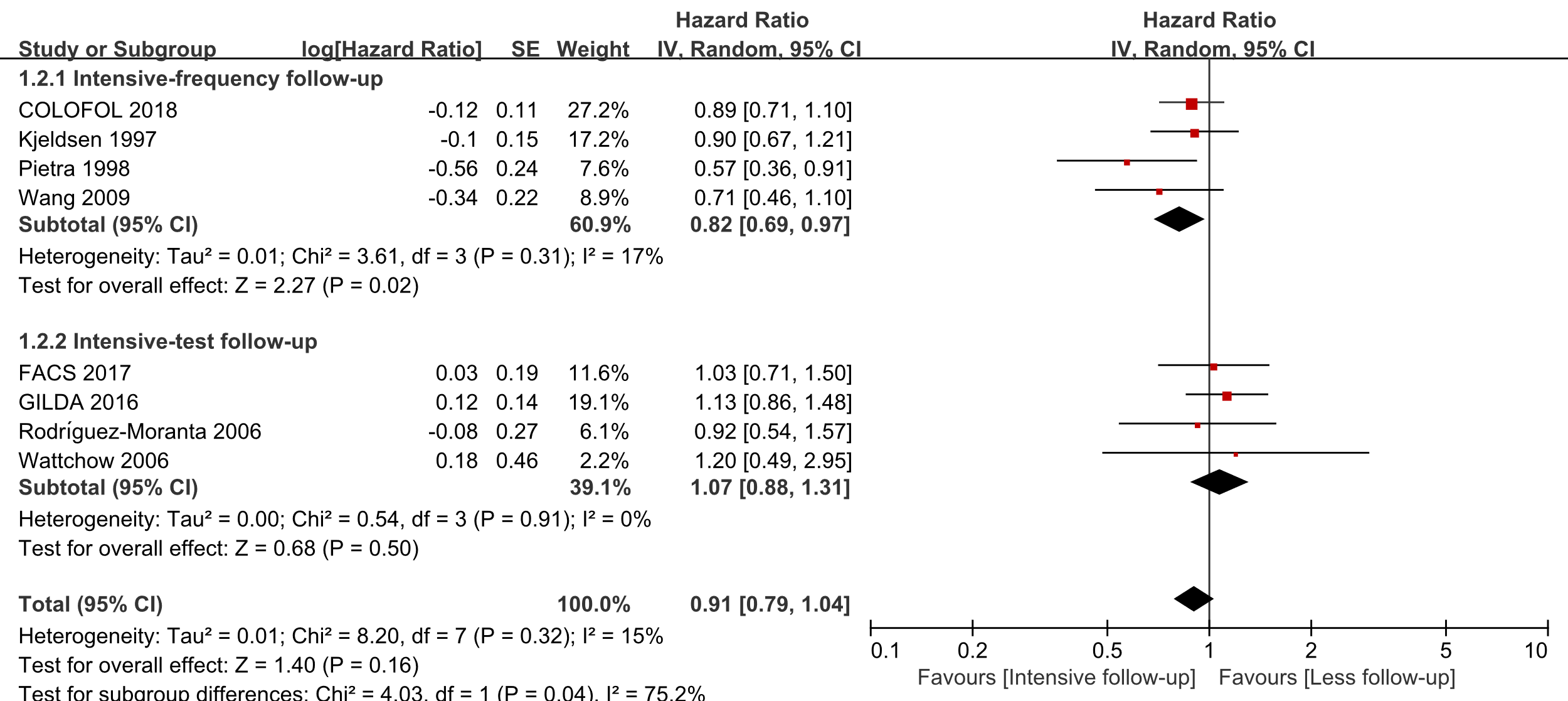


### Supplement eFigure 7: Subgroup analysis for overall survival –based on frequency of follow-up. df = degrees of freedom, M-H = Mantel-Haenszel.

###
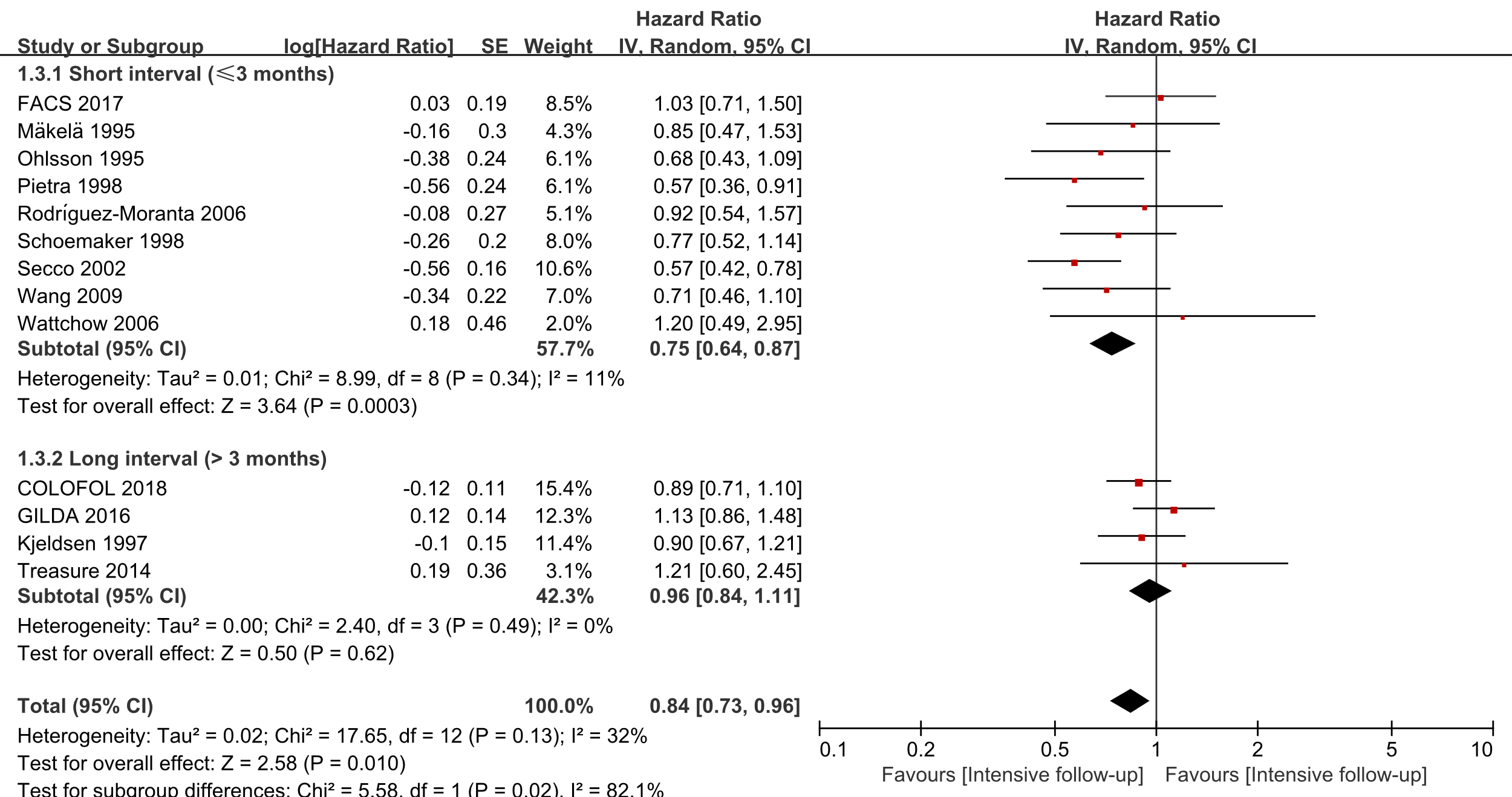

Supplement: S1 File — (DOCX) [file pone.0220533.s001.docx]
